# Hashimoto's encephalopathy – presenting with epilepsia partialis continua and a frontal lobe lesion

**DOI:** 10.1002/ccr3.1306

**Published:** 2017-12-05

**Authors:** Lokesh A. Rukmangadachar, Sudeepta Dandapat, Esther N. Bit‐Ivan, Yen‐Yi Peng

**Affiliations:** ^1^ Department of Neurology Southern Illinois University School of Medicine Springfield Illinois; ^2^ Department of Pathology Memorial Medical Center Springfield Illinois; ^3^ Department of Neurology Renown Institute for Neuroscience, Renown Health University of Nevada Reno Nevada 89502

**Keywords:** Epilepsia partialis continua, Hashimoto's encephalopathy, thyroglobulin antibody, thyroperoxidase antibody

## Abstract

We report a case of Hashimoto's encephalopathy (HE), who presented with epilepsia partialis continua (EPC) and a frontal lobe lesion. The diagnosis of HE remained elusive until the serum thyroid antibodies became positive 7 months after the onset of EPC. The histopathology of this frontal lesion showed nonvasculitic inflammation.

## Introduction

Hashimoto's encephalopathy (HE) has a protean clinical presentation and is often under‐recognized due to a lack of clinical suspicion and specific biomarkers. As Brain's initial publication of HE in 1966 1, HE has been reported to cause neurologic and neuropsychiatric manifestations such as seizure 2, epilepsia partialis continua (EPC) 2, 3, status epilepticus 4, tremor 2, myoclonus 2, stroke‐like symptom 2, dementia 5, stupor/coma 6, psychosis 7, headache 8, ataxia 9, and myelopathy 10. HE imaging findings are also varied. They include normal appearance, focal, or diffuse pathology. Tumor‐like lesions have been reported in some cases 2, 3, 11, 12. Elevated serum thyroperoxidase antibody (TPOAb) or elevated thyroglobulin antibody (TgAb) and encephalopathy reversible by steroid have been proposed to be necessary 13. However, when patients do not present in this classical way, diagnosis is often delayed and results in increased morbidity.

EPC in HE is rarely reported in the literature 2, 3 nor are tumor‐like lesions 2, 3, 11. Here, we report a patient of HE who presented with EPC and a contralateral frontal nodular lesion. This case is similar to the two patients reported earlier in the literature 2, 3. The cases differ in that total lesionectomy was performed and serum TPOAb and TgAb remained negative in the first 7 months of onset of EPC in this case.

## Case Report

We present a 49‐year‐old woman with a history of thyroidectomy 5 years prior to the onset of EPC. She was on thyroid replacement therapy. The pathological report was consistent with benign lymphoepithelial cysts of thyroid which could be associated with chronic thyroiditis occurring in a background of Hashimoto's or chronic lymphocytic thyroiditis 14.

She presented with a new onset of constant semirhythmic clonic twitching, weakness, and clumsiness in the left foot. The left foot clonic twitching persisted throughout the day (around once every 5 to 30 sec). At times, the clonic twitching spread to her left hand, leading to the contraction of her left fingers, wrist, and elbow; these episodes lasted around 30 sec. She had one witnessed generalized tonic‐clonic seizure (GTCS) 1 week after the onset of her left foot clonic twitching. The patient also had spells of grunting and posturing of the left limbs during sleep.

**Figure 1:**
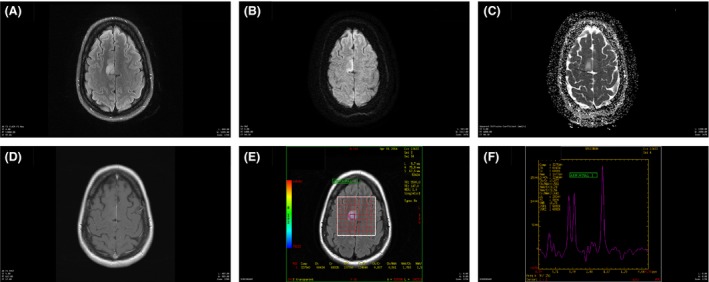
MRI of the brain. (A) T2 FLAIR sequence shows a hyperintense 17 mm × 14 mm nodular lesion within the right mesial frontal lobe. (B) Diffusion‐weighted imaging sequence shows increased signal without an ADC reduction, which suggests edema. (C) ADC map of the corresponding diffusion‐weighted image. (D) T1‐weighted postcontrast image shows no contrast enhancement. (E and F). Magnetic resonance spectroscopy of the lesion shows overall nonneoplastic spectrum.

**Figure 2:**
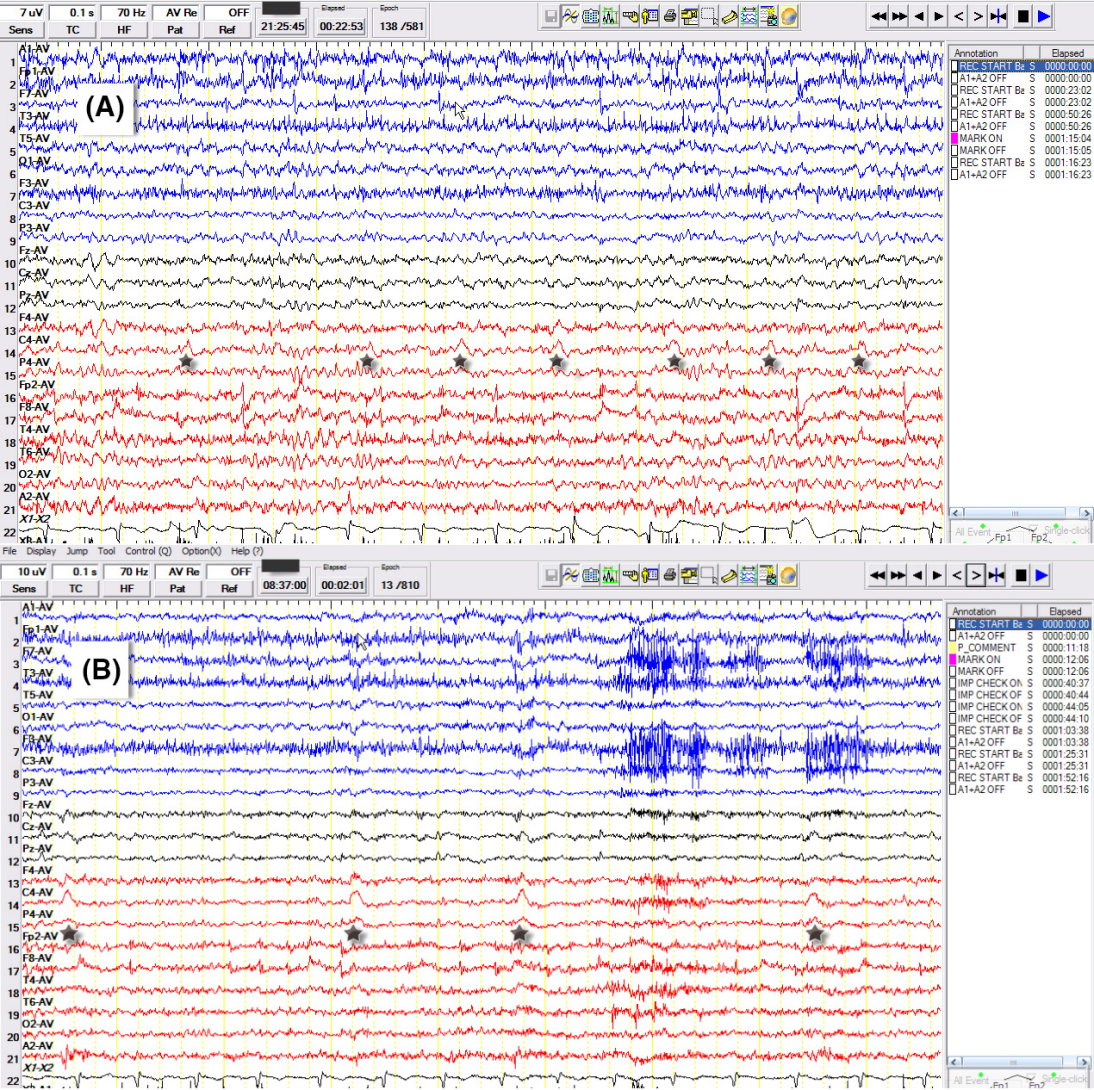
EEG. (A) Displayed on average reference montage, the long‐term video EEG showed recurrent, small‐amplitude, negative, blunted sharp waves (black stars), with the maximum over C4 lead. These sharp waves did not always precede the left foot twitching. These frontal sharp waves and the patient's jerking of the left limbs intensified after anti‐epileptic medications were discontinued. (B) Displayed on the average reference montage, the EEG was obtained on the following day of A, which showed recurrent sharp waves maximum over C4, P4, and Cz with higher amplitude compared with those of A. Of note, the sensitivity of B was decreased to 10 uV/mm. Eleven minutes after B, the patient developed a generalized tonic‐clonic seizure that was triggered by putting her left foot on the floor (reflex seizure).

The initial MRI of the brain was negative. The follow‐up MRI of the brain 2 weeks after the onset showed a nonenhancing lesion in the right mesial frontal gyrus. The lesion stayed stable in the subsequent 4 months (Fig. 1). Routine scalp EEG was unremarkable. Clonic twitching of the left foot decreased after trials of anti‐epileptic medicines, which included levetiracetam, oxcarbazepine, sodium valproate, and clonazepam. The video EEG monitoring revealed repetitive blunted sharp waves over the right central region (on the reference montage), which became prominent after the withdrawal of seizure medication. The video EEG evolved into one generalized tonic‐clonic seizure, which was triggered by standing on the floor (reflex seizure) (Fig. 2). The aura (sensation of falling) preceding the GTCS was a horrible experience for the patient so antiseizure medication was put back immediately after the first recorded GTCS. The repetitive blunted sharp waves over the right central region subsided after reinstitution of antiseizure medication. These blunted sharp waves could have been easily missed on the bipolar montage. Similarly, the epileptic clonic twitching could have been mistakenly classified as a spasm, tremor, or myoclonus. During this time, the patient remained awake and oriented.

**Figure 3:**
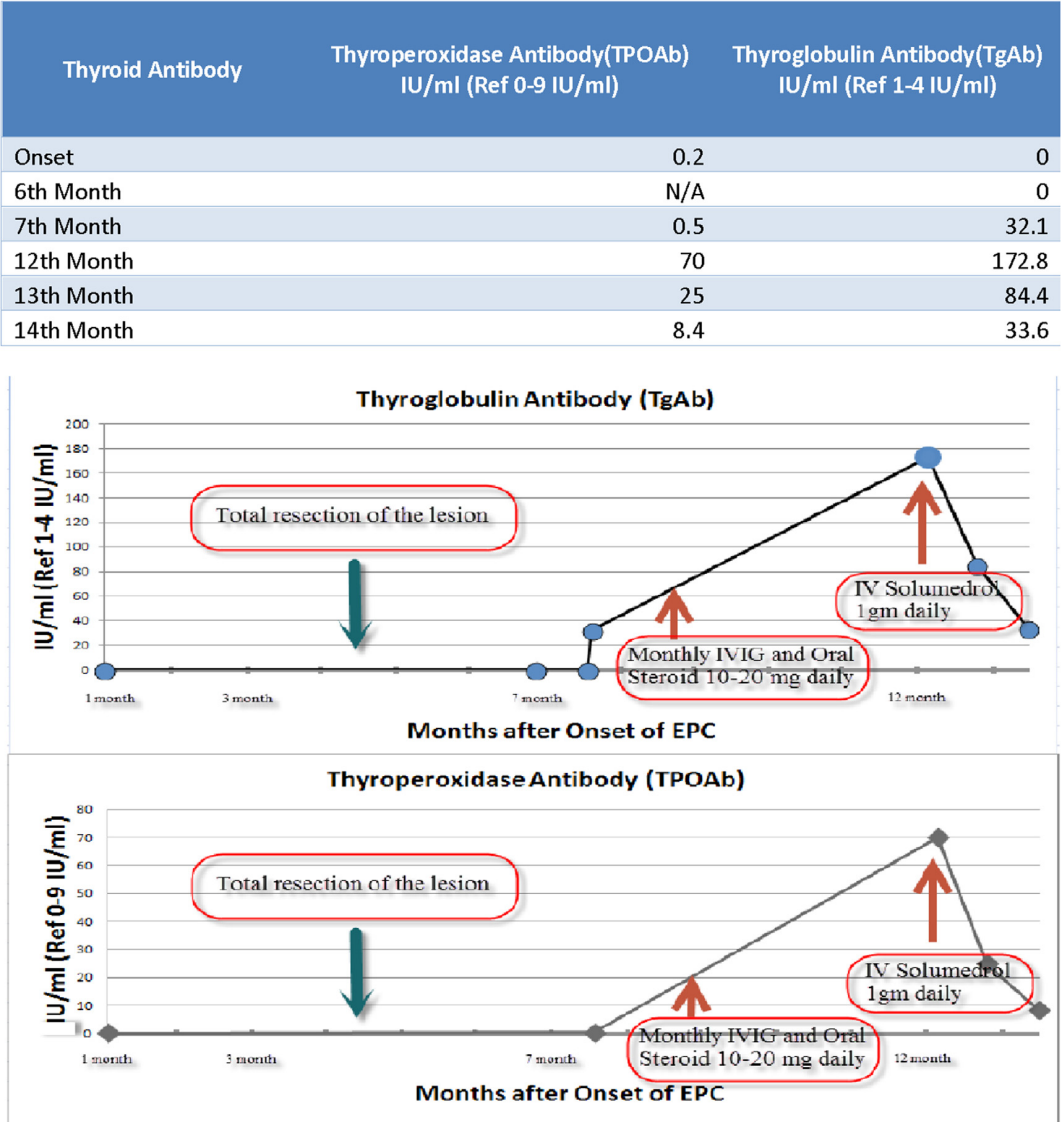
Serial change of serum TPOAb and TgAb.

The initial laboratory studies were not conclusive. The antinuclear antibody was 1:40 with a speckled pattern. The serum TPOAb and TgAb remained in the normal range during the first 7 months of EPC onset (Fig. 3). CBC, basic metabolic chemistry, thyroid functions (Free T3, Free T4, and Total T3) were within normal range. The CSF performed at the beginning had WBC 1 cells/uL RBC 164 cells/uL, Glucose 78 mg/dL, total protein 28 mg/dL, IgG CSF 15 mg/dL (reference range 0–6 mg/dL), IgG synthesis 30 mg/d (reference less than 8.0 mg/d), IgG index 0.1 (reference range 0.28–0.66), and myelin basic protein 5.98 mcg/L (reference range 0–4.0 mcg/L).

MRI of the entire spinal cord did not show any lesions, and the whole‐body computed tomography scan did not reveal any occult tumors.

**Figure 4:**
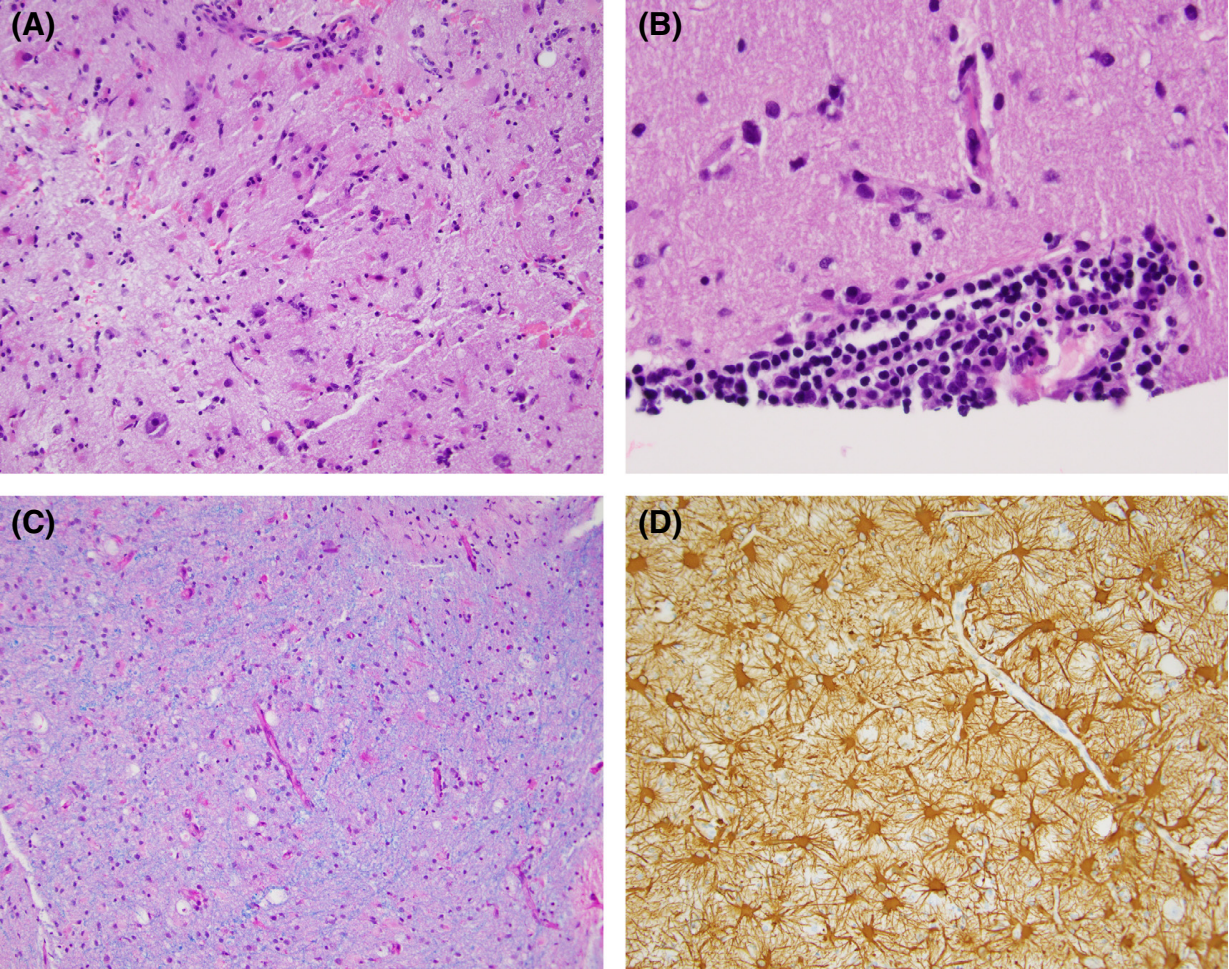
Histopathology. (A) Hematoxylin and eosin stain sections show the cortex with reactive astrocytes. (B) Perivascular lymphocytic infiltrate. (C) Luxol fast blue staining for myelin shows no significant myelin loss. (D) Immunohistochemistry with antiglial fibrillary acidic protein antibody highlights numerous reactive astrocytes. MIB 1 staining showed a proliferation index of less than 1%; p53 was negative (not shown). IDH1/2 mutation was not detected.

Four months after the onset of EPC, focal clonic seizures remained intractable. Repeated MRI of the brain showed the same frontal lobe lesion. Consciousness and cognition remained intact. The patient underwent a total resection of the lesion with the goal of controlling EPC. The lesion was found to be gliotic with marked perivascular and parenchymal inflammation (Fig. 4). This histopathology result did not support true vasculitis. Instead, it was consistent with nonvasculitic autoimmune inflammatory meningoencephalitis.

**Figure 5:**
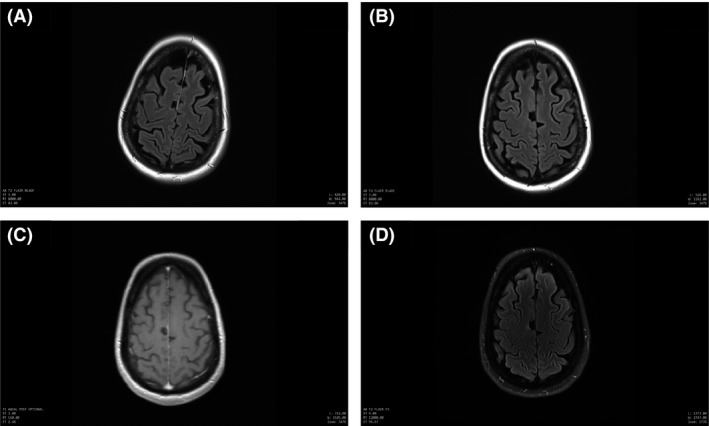
MRI of the brain after the lesionectomy. (A) Seven months after onset of EPC, T2 FLAIR sequence shows an area of encephalomalacia over the right frontal cortex in the region of surgery. (B) Twelve months after onset of EPC, T2 FLAIR sequence shows stable postsurgical changes over the right frontal cortex in the region of surgery despite the worsening of EPC. (C) Twelve months after onset of EPC, T1 postcontrast sequence shows no contrast enhancement around the area of encephalomalacia. (D) Two years after onset of EPC, T2 FLAIR sequence shows stable postsurgical changes. In addition, no asymmetric brain volume loss was noted.

After the lesionectomy, the left foot clonic twitching decreased temporarily. Six months after the onset of EPC, the clonic twitching of the left limbs worsened and spread to the ipsilateral face. At times, speech became unintelligible due to the ipsilateral tongue and facial clonic twitching. Consciousness and cognition remained intact. Although the EPC worsened after the lesionectomy, a follow‐up MRI of the brain 7 months after the onset did not show any new changes (Fig. 5).

Seven months after the onset, TgAb was mildly elevated at 32 IU/mL (Ref 1–4 IU/mL), but TPOAb remained within the normal range 0.5 IU/mL (Ref 0–9 IU/mL) (Fig. 3). The repeat CSF performed at the seventh month of onset had WBC 5 cells/uL RBC 14 cells/uL, Glucose 45 mg/dL, total protein 35 mg/dL, IgG CSF 15.6 mg/DL (reference range 0–6 mg/dL), IgG synthesis 61.2 mg/d (reference less than 8.0 mg/d), IgG index 2.8 (reference range 0.28–0.66), myelin basic protein 7.62 mcg/L (reference range 0–4.0 mcg/L), and positive oligoclonal bands 4 (reference range 0–1 unique CSF bands with no corresponding bands in the serum). CSF *N*‐methyl d‐aspartate receptor IgG antibody (NMDA) was negative. A panel for autoimmune encephalitis in serum (Athena Diagnostics, Inc. 200 Forest Street, 2nd Floor Marlborough, MA 01752) including anti‐Hu, NMDA receptor (NR1‐subunit), LGI1, anti‐GAD 65, anti‐VGKC, anti‐CASPR2, anti‐Ma1 and anti‐Ma2, anti‐CV2, and anti‐amphiphysin were all negative.

Under the working diagnosis of probable autoimmune encephalitis, the patient was started on monthly intravenous immunoglobulins (IVIG 2 g/kg) 9 months after the onset. There was a partial improvement of symptoms after monthly IVIG but the benefits were temporary. Low‐dose steroids – prednisone (10 mg–20 mg daily) were added but they failed to halt the EPC progression (Fig. 3). Rasmussen encephalitis or mild forms of HE were probable diagnoses.

Twelve months after the onset of EPC and 3 months after the IVIG treatment, the patient developed poor appetite, disorientation, and confusion. The patient was admitted to the hospital. MRI of the brain showed stable postsurgical changes (Fig. 5) but serum TPOAb and TgAb were elevated further to 70 IU/mL and 172 IU/mL (Fig. 3), respectively. Therefore, the patient was administered high‐dose IV methylprednisolone 1000 mg daily for 3 days. Consciousness and appetite improved dramatically, and the patient was prescribed oral 60 mg prednisone daily after discharge.

Fourteen months after the onset of EPC, the patient's mental status normalized, left limb twitching decreased, and speech became intelligible. With steroid treatment, clinical improvement was accompanied by a parallel decrease in the serum TPOAb and TgAb levels (Fig. 3).

## Discussion

This case of HE presented with EPC and contralateral frontal lobe lesion. It took 1 year for this patient to develop steroid‐responsive encephalopathy. Finally, the case met the traditional diagnostic criteria of HE despite serum thyroid antibodies that remained negative in the first 7 months. Lesionectomy was performed, and histopathology showed nonvasculitic inflammation. The progressive clinical course of this case suggests that HE is an autoimmune spectrum disorder.

This patient is unique in the following three aspects.

First, the patient was not encephalopathic, and serum thyroid antibodies remained negative during the first 7 months of EPC. Our team did not start immunotherapy immediately. The authors hypothesize that the traditional diagnostic criterion of HE – steroid‐responsive encephalopathy manifesting as cognitive impairment – only represents the severe forms of the HE spectrum disorder.

Second, before the thyroid antibodies turned positive, the patient underwent a lesionectomy. Pathological reports of HE have been few in the literature, and their results were not consistent 5, 11, 13, 15, 16, 17, 18, 19, 20, 21, 22. This case's pathological report showed nonvasculitic autoimmune inflammatory meningoencephalitis instead of true vasculitis 23.

Lastly, this case presented with constant semirhythmic left foot twitching (a focal motor seizure affecting the left foot associated with a contralateral frontal cortex lesion). Could HE have its preference regarding the areas of the brain it affects?

Based on the published case series 13, 24, the protean clinical manifestations of HE include seizures, multiple stroke‐like episodes, tremor, and myoclonus. The stroke‐like episodes, tremor, and myoclonus in HE could represent occult seizure originating from the motor/sensory cortex.

Nonspecific EEG abnormalities such as diffuse slowing were well documented in HE 2, 25. Because scalp EEG is not sensitive in detecting frontal lobe seizures, nonspecific EEG abnormality suggests that HE may have a preference in affecting the motor/sensory cortex close to the frontal lobe.

As the prevalence of thyroid antibodies is high in the general population, the link between thyroid antibodies and the neurological syndrome was regarded to be spurious in the asymptomatic population 26. On the other hand, it is also possible that mild forms of HE are underdiagnosed because its clinical manifestations such as tremor, tingling, twitching, psychosis, or stroke‐like symptoms are transient, vague, and at times hard to differentiate from psychogenic disorders. Better objective tests would be necessary to clarify this issue.

In the last decade, autoimmune epilepsy has become increasingly recognized to be one of the major causes of seizures of unknown etiology (more than 20%) 27. In one study, thyroid antibodies were observed more commonly in females with late‐onset focal epilepsy with unknown etiology (7.8%) 28.

HE, as our case illustrates, can present as progressive EPC with a focal cortical lesion. It is important to consider this diagnosis in any patient with intractable EPC and/or progressive encephalopathy. Initial normal thyroid antibody levels do not completely exclude the diagnosis of HE. HE could be underdiagnosed as the currently accepted diagnostic criteria of HE represent only the severe cases of HE spectrum disorder.

## Authorship

LAR and SD: assisted in coordinating the patient's care and writing this manuscript. ENBI: was the pathologist for this patient. YYP: was the neurologist for this patient.

## Conflict of Interest

All authors declare no conflict of interest.
